# The Prognostic Significance of BRAF Gene Analysis in Children and Adolescents with Papillary Thyroid Carcinoma: A Systematic Review and Meta-Analysis

**DOI:** 10.3390/diagnostics13061187

**Published:** 2023-03-21

**Authors:** Eleni P Kotanidou, Styliani Giza, Vasiliki Rengina Tsinopoulou, Kosmas Margaritis, Anastasia Papadopoulou, Eleni Sakellari, Savvas Kolanis, Eleni Litou, Anastasios Serbis, Assimina Galli-Tsinopoulou

**Affiliations:** 1 Unit of Pediatric Endocrinology and Metabolism, 2nd Department of Pediatrics, School of Medicine, Faculty of Health Sciences, Aristotle University of Thessaloniki, AHEPA University Hospital, Stilponos Kyriakidi 1, 54636 Thessaloniki, Greece; 2 Department of Pediatrics, School of Medicine, Faculty of Health Sciences, University of Ioannina, 45500 Ioannina, Greece

**Keywords:** children, adolescents, papillary thyroid cancer, proto-oncogene B-raf gene (BRAF), prognosis

## Abstract

Thyroid cancer represents the prominent endocrine cancer in children. Papillary thyroid cancer (PTC) constitutes its most frequent (>90%) pediatric histological type. Mutations energizing the mitogen-activated-protein kinase (MAPK) pathway are definitely related to PTC. Its most common genetic alteration is in proto-oncogene B-Raf (BRAF). Mutated BRAF is proposed as a prognostic tool in adult PTC. We conducted a systematic review and meta-analysis evaluating the association of mutated BRAF gene and prognostic clinicopathological characteristics of PTC in children/adolescents. Systematic search for relevant studies included PubMed, MEDLINE, Scopus, clinicaltrials.gov and Cochrane Library. Pooled estimates of odds ratios for categorical data and mean difference for continuous outcomes were calculated using random/fixed-effect meta-analytic models. BRAFV600E mutation presents a pooled pediatric/adolescent prevalence of 33.12%. Distant metastasis is significantly associated with mutated BRAF gene (OR = 0.32, 95% CI = 0.16–0.61, *p* = 0.001). Tumor size (MD = −0.24, 95% CI = −0.62–0.135, *p* = 0.21), multifocality (OR = 1.13, 95% CI = 0.65–2.34, *p* = 0.74), vascular invasion (OR = 1.17, 95% CI = 0.67–2.05, *p* = 0.57), lymph node metastasis (OR = 0.92, 95% CI = 0.63–1.33, *p* = 0.66), extra-thyroid extension (OR = 0.78, 95% CI = 0.53–1.13, *p* = 0.19) and tumor recurrence (OR = 1.66, 95% CI = 0.68–4.21, *p* = 0.376) presented no association or risk with BRAF mutation among pediatric/adolescent PTC. Mutated BRAF gene in children and adolescents is less common than in adults. Mutation in BRAF relates significantly to distant metastasis among children/adolescents with PTC.

## 1. Introduction

Thyroid cancer represents the principal endocrine cancer in pediatric and adolescent population, with a female predominance of 4:1 [1]. Although rare, accounting for about 4% of pediatric malignancies, a rapid increase in incidence has been documented, almost globally [2]. The most common histological type recognized in >90% of cases is papillary thyroid cancer (PTC) [3].

It has long been supported that pediatric and adult patients with thyroid cancer have distinct characteristics in terms of initial presentation, clinical course, and mortality. Children and adolescents are often diagnosed with more advanced disease, exhibiting an increased rate of lymph node and distant metastases and often have persistent or recurrent disease [4]. Paradoxically, the prognosis is more favorable in children than in adults, as evidenced by the high overall survival rate of 97.70% from 1975 to 2005, which has been further advanced to 99.27% from 2006 to 2016, according to the Surveillance, Epidemiology and End Results (SEER) database [5].

This phenomenon may be explained on a molecular basis, as the two populations also have different genetic features. Point mutations activating the mitogen-activated protein kinase (MAPK) pathway play an important role. Among them, the most common genetic alteration of PTC in adults is located in the proto-oncogene B-Raf (BRAF) gene and consists of a T to A transversion (T1799A), resulting in a valine to glutamate substitution at residue 600 (V600E) of the BRAF protein. However, recorded mutation prevalence rates range from 27% to 83% among different populations [4]. Since there is a lack of definitive evidence on the clinicopathological significance of BRAF V600E in adult PTC, several meta-analyses have been conducted to elucidate its role in the diagnosis, management and prognosis of aggressive PTC cases. Various associations have been reported between the presence of BRAF V600E mutation and demographic data or risk factors, such as tumor size, multifocality, lymph node metastasis, vascular invasion, extra-thyroid extension, and advanced stage of tumor node metastasis [6,7,8,9,10].

In the pediatric population, BRAF V600E, although prevalent, is recognized at a lower rate compared to adults. In sporadic pediatric PTC, the BRAF V600E mutation ranged between 0 and 63% in different studies [11,12,13,14,15,16,17,18,19]. Furthermore, the BRAF V600E mutation is not clearly associated with distinct negative clinicopathological features and does not predict an unfavourable course, in contrast to adult PTC [12,13,18,20]. In the recently published European Thyroid Association Guidelines for the management of pediatric thyroid nodules and differentiated thyroid cancer (DTC), the authors recommend that the molecular gene analysis for the presence of BRAF V600E mutation in a fine-needle aspiration (FNA) specimen may be useful for diagnosis of PTC and therefore may be incorporated into the diagnostic work-up [21]. However, the use of BRAFV600E as a molecular marker in pediatric and adolescent PTC remains controversial.

Mutant BRAF is proposed to serve as a diagnostic and prognostic tool and may be a promising target for molecular therapy [22]. The real incidence and evidence of the actual effect of BRAF mutation in pediatric PTCs remains controversial in different studies. In this context, we conducted a systematic review of available evidence and a meta-analysis of data published over the past two decades to evaluate the association of BRAF gene mutations and PTC in children and adolescents, their prognostic role in terms of clinicopathological characteristics, and relationship with survival outcome.

## 2. Materials and Methods

The current systematic review and meta-analysis was designed following a predefined protocol, according to the Preferred Reporting Items for Systematic Reviews and Meta-Analysis (PRISMA) guidelines, which is registered in the PROSPERO database under the identification number: PROSPERO 2022 CRD42022358663.

### 2.1. Eligibility Criteria 

The present systematic review included all original studies that reported a molecular study of proto-oncogene BRAF gene, in children and adolescents, aged up to 21 years, with a histopathological diagnosis of PTC. Studies were included only if they reported clinical or laboratory characteristics of PTC and/or assessed the overall survival of their participants. According to our predefined eligibility criteria, the diagnosis of PTC needed to be confirmed in all included individuals by either tumor biopsy or FNA biopsy, while BRAF genetic analysis could be reported by any available molecular method, such as Sanger analysis or direct sequencing. Relevant studies published in the last 20 years were identified. Language was restricted to English. No limitation of publication status was implied. 

Exclusion criteria, in order to minimize potential publication bias and duplication of results, referred to studies including patients older than 21 years of age, data from univariate analyses if the HR was the primary outcome, studies involving other than PTC carcinomas, review articles, case reports, presentations, conference proceedings, editorials, expert opinions, research using big data (e.g., using SEER study data) and in vitro studies.

### 2.2. Study Outcomes

The main outcome of the present study was to investigate the difference in the prevalence of known prognostic factors between children and adolescents with PTC and a mutated BRAF gene compared to PTC patients without BRAF gene alterations. More specifically, the primary outcomes combined differences in the prevalence of the following tumor variables: multifocality, vascular invasion, extra-thyroid extension (ETE), presence of lymph node metastasis (LNM), distant metastases, and tumor recurrence. Data on differences in diametric tumor size and data regarding gender (male/female) distribution were also compared between BRAF positive and negative patients. The secondary outcome was the absolute overall survival difference of PTC patients with BRAF gene mutations compared to PTC patients without BRAF gene mutations.

### 2.3. Information Sources

Relevant studies over the past 20 years, evaluating the association of BRAF mutations with prognostic factors for PTC in children and adolescents, were identified by searching the following databases: PubMed, Ovid Medical Literature Analysis and Retrieval System Online (MEDLINE), SCOPUS, the US registry of clinical trials [www.clinicaltrials.com (accessed on 30 September 2022)] Cochrane Central Register of Controlled Trials and Cochrane reviews. Search was performed on September 2022, using a combination of relevant terms in the English language, such as “BRAF gene”, “B-raf gene”, “proto-oncogene B-raf gene”, “papillary thyroid cancer”, “children”, “adolescents”, “young adults”. Two reviewers independently selected studies according to the inclusion criteria, while a third independent reviewer was available to address any discrepancies. Bibliographies from review articles were thoroughly examined to identify relevant studies, ensuring that papers and articles not selected in the initial search were also included. 

### 2.4. Screening, Data Collection and Analysis

Conducted with a pilot-tested form by two reviewers and verified by a third using a predefined datasheet, data collection was performed. Two authors, E.P.K. and S.G., with expertise in systematic review, screened all titles and abstracts for eligibility, in a completely independent manner. Full texts were reviewed by the two reviewers and discrepancies were resolved with the involvement of a third reviewer, A.G.T. Reasons for exclusion were recorded for all studies excluded in the title, abstract or full text level of the review process. Data were extracted from full texts of the studies on a predefined worksheet. Two authors (A.P. and E.L.) extracted the following from the included articles: first author, country, publication years, study type, recruitment period, sample size, sample origin, method of BRAF analysis, BRAF V600E mutations, PTC-related risk factors. Age, gender, tumor size, vascular invasion, LNM, ETE, lymph node metastasis and distant metastasis were concluded as the predefined risk factors for PTC patients. The survival rate after any timepoint in retrospective, or in the present in prospective, studies was also recorded. Finally, funding sources and authors’ conflict of interest were recorded and included. Any disagreements were resolved by a third investigator (E.P.K.).

### 2.5. Quality Assessment of Included Studies 

Quality assessment of the included studies was conducted by two reviewers (E. S. and V.R.T.) using the Critical Appraisal Checklist JBI Tool for Analytical Cross-Sectional Studies, developed by the JBI, Faculty of Health and Medical Sciences at the University of Adelaide [23]. The tool consists of eight different questions that assess the methodological quality of each study, determining the extent to which the possibility of bias has been addressed in its design, conduction and analysis. Each question was rated by the two reviewers as green for “Yes”, red for “No”, or yellow for “Unclear”. Discrepancies were resolved by discussion.

### 2.6. Measures of Effect

Effect measures used in the synthesis and presentation of results were set as follows: continuous outcomes as mean difference and 95% CI; dichotomous outcomes as odds ratio and 95% CI.

### 2.7. Data Synthesis

Data synthesis was performed with random-effects model, and two-tailed statistical significance was defined as a *p*-value < 0.05. For statistical analysis, the Comprehensive Meta-Analysis, v3.0 software (Biostat, Englewood, NJ, USA), was employed. The magnitude of the effect of each study was calculated by the OR, or briefly by the weighted mean difference (WMD) of the 95% CI briefly. In addition, heterogeneity was quantified using the I^2^ statistic. When I^2^ < 50%, a fixed-effects model was applied; otherwise, a random-effects model was used. The Begg funnel plot was used to control for potential publication bias.

## 3. Results

### 3.1. Study Selection and Characteristics

**Figure 1:**
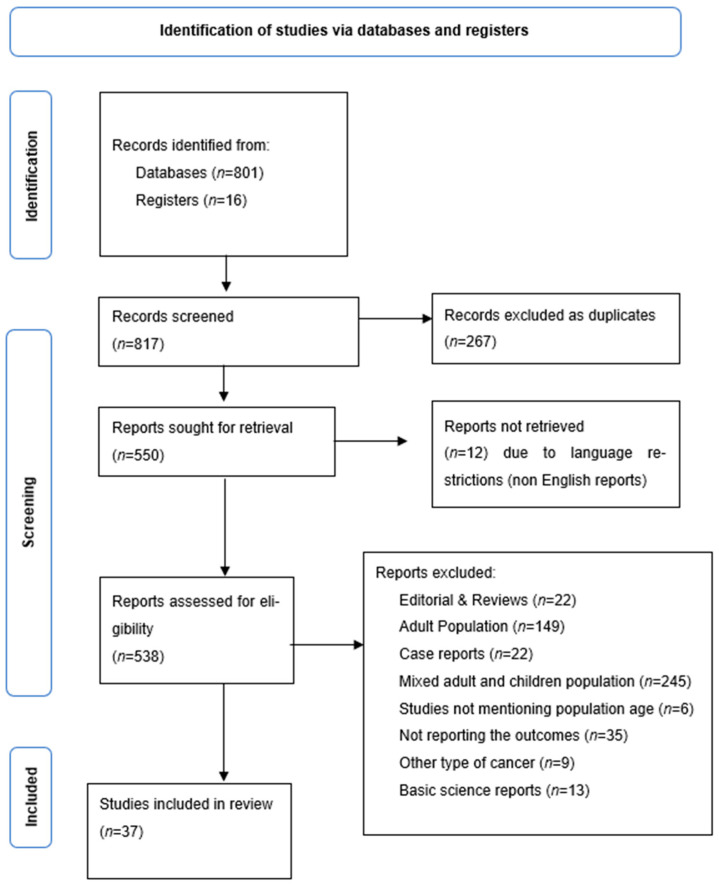
Systematic review flow chart of records identification and study screening.

The initial systematic screening of the available evidence resulted in a total of 817 studies. Among them, 267 records were excluded as duplicates; 12 records were excluded due to language other than English; 22 records were excluded as reviews and editorials; 22 records were excluded as case reports; 149 records were excluded as they concerned adult populations; 245 records were excluded since they reported mixed adult and child/adolescent population data, without subgroup analyses; six records were excluded since they did not mention the exact age of the population; 35 records were excluded as they did not report any of the outcomes of the present review; nine records were excluded due to populations with cancer type other than PTC; 13 records were excluded as basic science studies. Finally, a total of 37 studies that met our selection criteria were included in our meta-analysis. The selection flowchart of the research is presented in Figure 1. 

Basic characteristics of the included studies and the associated factors examined are included in Table 1. Data regarding the methodology applied in the molecular analysis of the BRAF gene in included studies are presented in Supplementary Table S1, in combination with details regarding funding of the studies. 

### 3.2. Prevalence of BRAF Mutation 

The most prevalent mutation of BRAF gene molecular analyses across all studies was BRAF V600E mutation, with reported prevalence rates ranging from 0% to 64.2% among pediatric and adolescent populations. Other reported genetic alterations of BRAF gene were BRAF c.1799_1801delTGA, BRAF T1796A, BRAF T599del, BRAF K599I, and BRAF K601E, with extreme modest frequency rates compared to V600E (Table 1). 

Overall, BRAFV600E mutation was confirmed among a group of 596 individuals from a totality of 1799 children and adolescents with PTC in this systematic review and meta- analysis, resulting in a pooled BRAFV600E prevalence of 33.12% (Table 1 and Table 2).

### 3.3. BRAF Mutation and Gender

**Figure 2:**
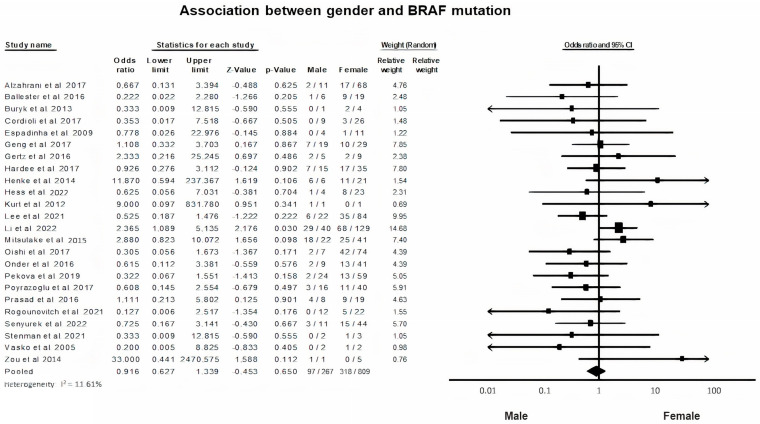
BRAF mutation and Gender correlation forest plot [7,12,14,15,16,17,18,20,24,25,26,28,30,31,33,37,38,40,42,43,45,47,48,49].

A random-effects model was applied to analyze the data of relevance among mutated BRAF and gender (*p* = 0.65, I^2^ = 11.6%). Prevalence of BRAFV600E mutation in female PTC patients was relatively higher than that in male PTC patients, without reaching significance (OR = 0.91, 95% CI = 0.62–1.33) (Figure 2).

### 3.4. BRAF Mutation and Tumor Size

**Figure 3:**
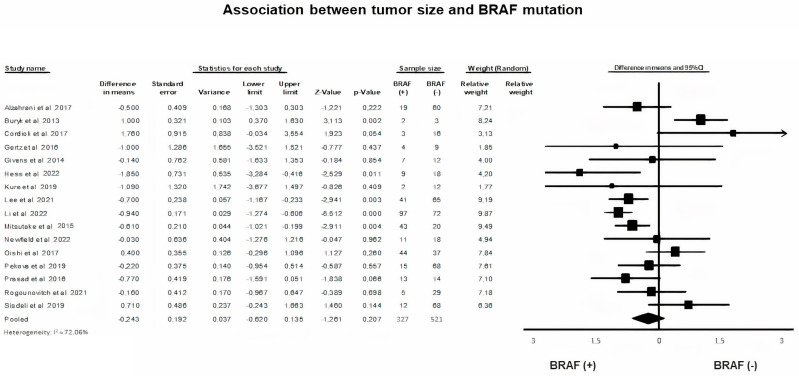
BRAF mutation and tumor size forest plot [7,13,14,15,16,17,25,28,29,31,33,35,37,40,43,46].

A random-effects model on continuous data was applied to explore the effect of the presence of BRAF mutation to the size of PTC as expressed by the actual tumor diameter (*p* = 0.21, I^2^ = 72.06%). Meta-analysis revealed that tumor size was not significantly associated with BRAF mutation in children and adolescent patients with PTC (Mean Difference = −0.24, 95% CI = −0.62–0.135, St. error = 0.192) (Figure 3).

### 3.5. BRAF Mutation and Multifocality

**Figure 4:**
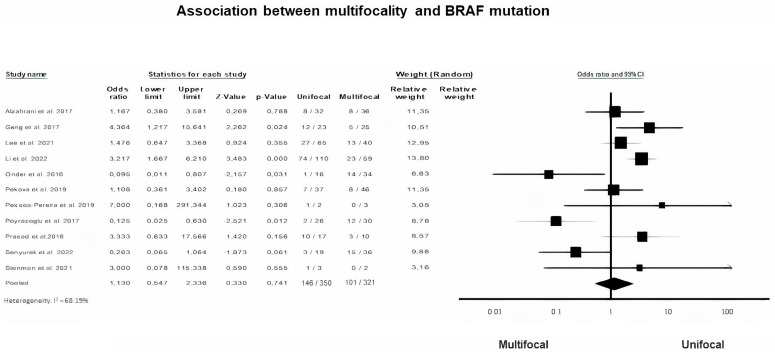
BRAF mutation and Multifocality forest plot [7,14,17,18,31,38,40,41,42,45,47].

A random-effects model was applied to analyze dichotomous data on the presence of multifocality in PTC (*p* = 0.74, I^2^ = 68.19%). According to our findings, tumor multifocality was not associated with BRAF gene mutation in pediatric and adolescent PTC (Table 2, OR = 1.13, 95% CI = 0.65–2.34) (Figure 4).

### 3.6. BRAF Mutation and Vascular Invasion 

**Figure 5:**
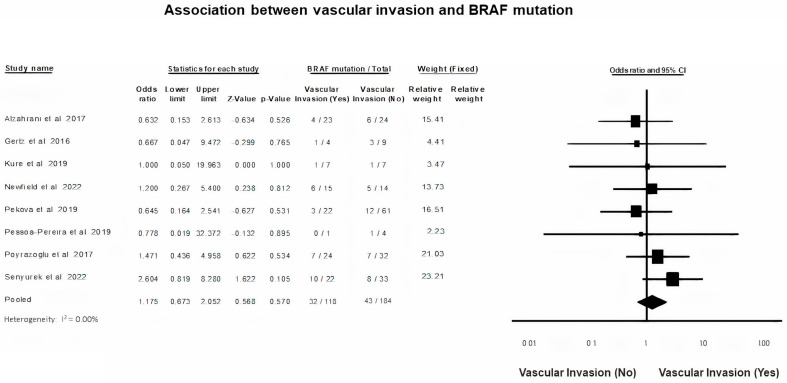
BRAF mutation and Vascular invasion forest plot [15,17,29,35,40,41,42,45].

A fixed-effects model was applied to analyze the presence of vascular invasion in PTC (*p* = 0.57, I^2^ = 0%). Pooled data of the present meta-analysis prove that the presence of vascular invasion in children and adolescents with PTC, does not exert a significantly higher risk for BRAF mutation (OR = 1.17, 95% CI = 0.67–2.05) (Figure 5).

### 3.7. BRAF Mutation and Lymph Node Metastasis (LNM)

**Figure 6:**
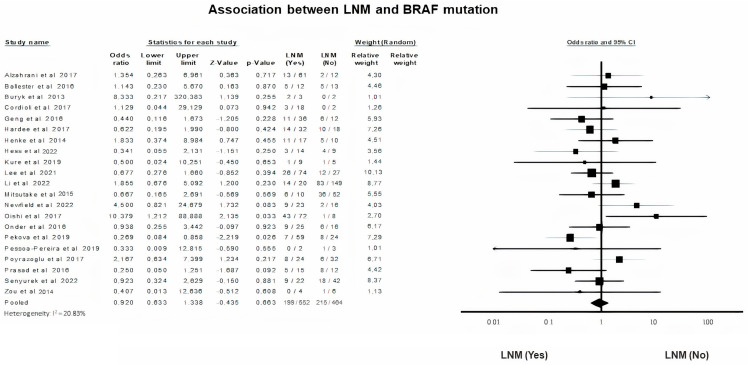
BRAF mutation and LNM forest plot [7,12,14,16,17,18,20,24,25,28,29,31,33,35,37,38,40,41,42,45,49].

Data on the presence or not of Lymph Node Metastasis (LNM) upon diagnosis of PTC in children and adolescents was analyzed after the application of a random-effects model (*p* = 0.66, I^2^ = 20.83%). LNM is not associated with mutated or absence of BRAF in PTC children and adolescents (OR = 0.92, 95% CI = 0.63–1.33) (Figure 6).

### 3.8. BRAF Mutation and Extrathyroidal Extension (ETE)

**Figure 7:**
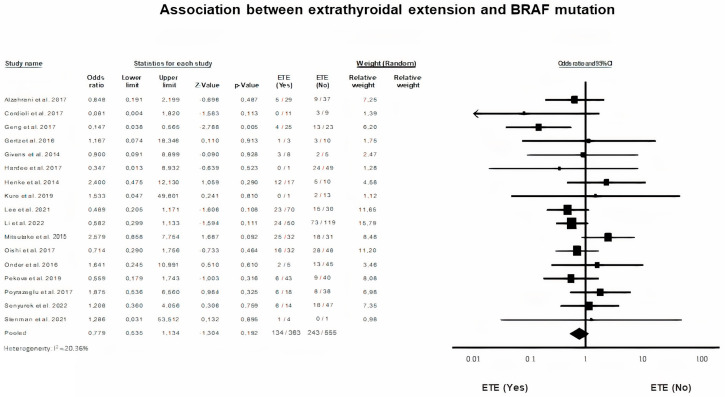
BRAF mutation and ETE forest plot [7,12,13,15,16,17,18,20,29,31,33,37,38,40,42,45,47].

A random-effects model was applied in order to meta-analyze categorical data on the presence or not of extrathyroidal extension of the PTC tumor in our study pooled population (*p* = 0.19, I^2^ = 20.36%). ETE is not significantly related to a higher rate of carrying a mutated BRAF gene among pediatric and adolescent patients with PTC (OR = 0.78, 95% CI = 0.53–1.13) (Figure 7).

### 3.9. Distant Metastasis in BRAF Mutation

**Figure 8:**
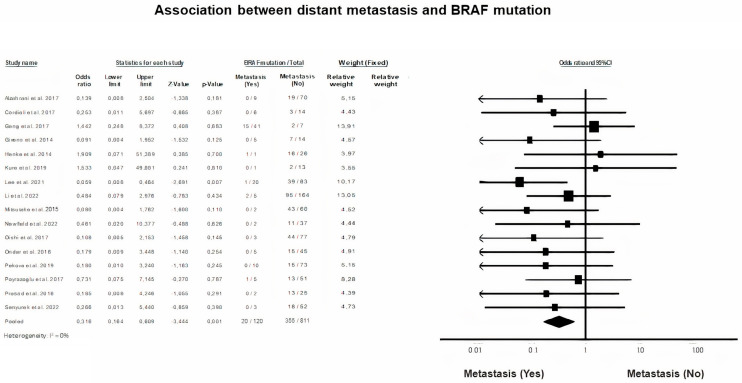
BRAF mutation and Distant Metastasis Forest plot [7,12,13,14,16,17,18,29,31,33,35,37,38,40,42,45].

A fixed-effects model was selected to analyze the correlation between the presence of BRAF mutation and the emerge of distant metastasis after PTC (*p* = 0.001, I^2^ = 0%). It was found that distant metastasis is significantly associated with the presence of a mutated BRAF gene in children and adolescents with PTC (OR = 0.32, 95% CI = 0.16–0.61) (Figure 8).

### 3.10. Tumor Recurrence and BRAF Mutation

**Figure 9:**
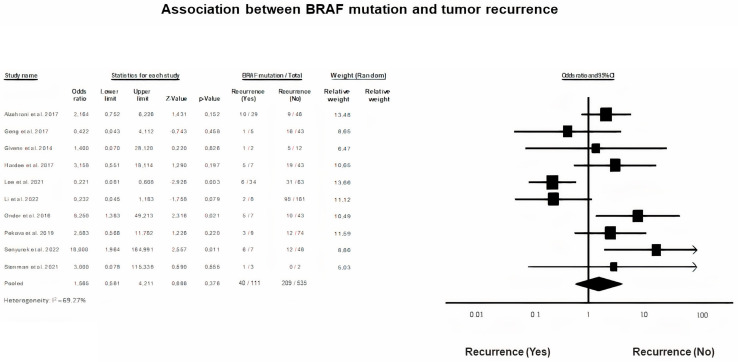
BRAF mutation and Tumor Recurrence Forest plot [7,13,17,18,20,31,38,40,45,47].

A random-effects model was utilized to analyze data regarding tumor recurrence rates and the presence of a mutated BRAF gene in children and adolescents with PTC (*p* = 0.376, I^2^ = 69.27%). It was found that BRAF mutation is not associated with tumor recurrence in the studied population (OR = 1.66, 95% CI = 0.68–4.21) (Figure 9).

### 3.11. BRAF Mutation and Survival Rate

Survival rate appeared as a variable reported in very few pediatric and adolescent studies. According to the present analyses, only a few records were identified as measuring survival at different time-points. Nies et al. report an overall 5-year survival rate of 98.5% in their small BRAF mutated cohort group [36]. In the same study, follow-up time ranged from 0.8–65 years, and thus authors report a 20-, 25-, and 30-year overall survival rate at 93.5%, 90.6%, and 86.8%, respectively [36]. Hardee et al. report a 100% survival rate in their cohort, with a follow- up time frame ranging from 10 to 42 years, as they recorded survival in year 2015 [20]. Mollen et al. reported a 100% survival rate in a median follow-up time of 6-years [34]. Finally, Henke et al. also report 100% overall survival during a 13.4 year study period [12]. It is obvious that, in pediatric protocols, recording survival is of poor scientific interest and is largely expected to reach the maximum (100%). The ten-year survival rate, usually reported in adult oncology, constitutes an outcome that was fairly reported in the childhood or adolescent cohorts, reflecting its difficulty in interpretation. 

### 3.12. Assessment of Quality and Biases of the Included Studies

**Figure 10:**
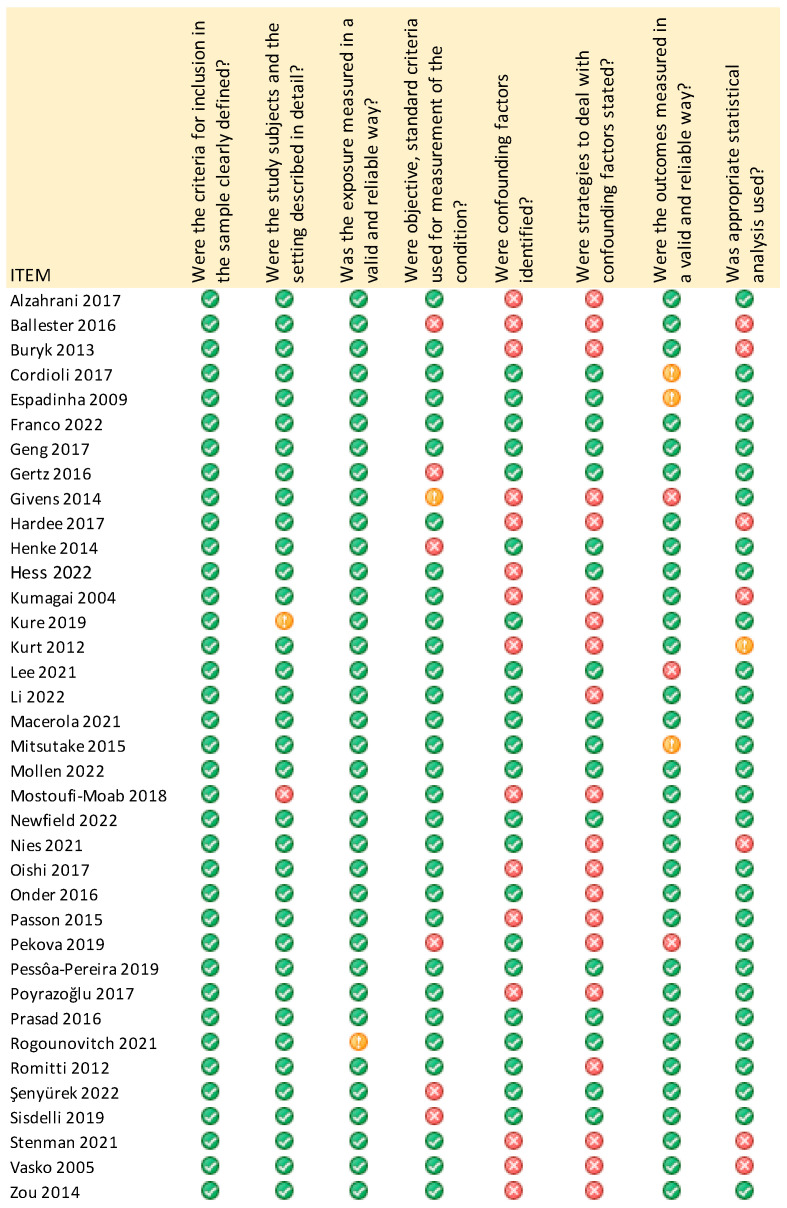
Risk of bias summary; authors’ judgements on each risk of bias item for each included study [7,11,12,13,14,15,16,17,18,19,20,24,25,26,27,28,29,30,31,32,33,34,35,36,37,38,39,40,41,42,43,44,45,46,47,48,49].

Quality assessment of included studies was performed using the JBI tool, consisting of eight different items, each of which scored 1 if the statement was ‘Yes’ (green sign) and 0 if the statement was ‘No’ (red sign), or ‘Unclear’ or ‘Not applicable’ (yellow sign) (Figure 10). The vast majority of studies assessed were considered as high quality (54.5%). More precisely, 35.13% scored seven out of eight points and 18.91% were graded with eight out of eight points, based upon the JBI quality assessment tool. Fifteen studies out of thirty-seven were at moderate risk related to quality assessment (40.53%), scoring five out of eight points (21.62%) and six out of eight (18.91%). Only a small percentage of studies (5.4%) recorded as low quality [13,24], since they confronted issues with domains regarding the criteria used for measurement of condition, identification of confounding factors and strategies to deal with them.

The domain “inclusion criteria definition” was the only item of the JBI tool which all studies succeeded in scoring. Most of the studies met difficulties in scoring the items “identification of confounding factors” and “strategies statement to deal with cofounding factors”. Accordingly, the least scored domain was “strategies to deal with confounding factors”, in which 20 out of 37 studies scored zero (54%).

## 4. Discussion

The discrete clinical behaviour of pediatric and adult PTC seems also to derive from distinct differences at the molecular level. An essential factor promoting the factor of tumorigenesis is the activation of the MAPK pathway through genetic alterations of its components. Among them, BRAF gene holds a key role, almost exclusively through the V600E mutation, which is recognized as the most frequent oncogenic variant in adult PTC. Due to its high prevalence, BRAF V600E has gained special research interest as to whether it could serve to identify patients with a potential for an aggressive clinical course. This research query has been extensively explored by meta-analyses of studies concerning adult PTC cases [6,7,8,9,10]. The present study consists of the first coordinated attempt to systematically review and meta-analyse all available evidence on pediatric and/or adolescent PTC, in order to elucidate any association of BRAF gene mutations with the clinicopathological features and the long-term outcome in the age of interest (<21 years). Except for the profoundly lower frequency of BRAF V600E in children and adolescents with PTC compared to adults, only distant metastasis upon diagnosis was as unfavourable prognostic factors that was associated with the BRAF V600E mutation. Furthermore, the excellent prognosis can only be hypothesized, due to the scarce data on survival rate, not allowing further analysis.

The mutation of great interest in the literature was BRAF V600E, which has consistently emerged as the most prevalent alteration of the BRAF gene. The predominance of V600E mutation in the longitudinal analyses of BRAF is recorded both during the first years of the specific loci study, two decades ago, through classical molecular techniques [11], and recently through advanced techniques such as high-resolution melt analysis [32] and next generation sequencing [27,35]. The widely varying prevalence from 0% [39] to 64.2% [33] in different protocols, resulted in a pooled prevalence of 33.12% among individuals < 21 years of age. Thus, it is shown that pediatric cohorts exhibit a lower frequency of BRAF V600E mutation frequency than adult series, where the overall estimated prevalence was 74.63% [10], almost 2-fold more, ranging between 25.4–89% [50,51]. The great difference in mutation rates among studies may be attributed to the variable heterogenous proportion of children and adolescents included. Two studies found that patients with BRAFV600E mutation positive tumors were significantly older than the BRAF V600E negative patients [20,46]. In contrast, Geng et al. [18] reported that the presence of BRAFV600E mutation was associated with age at diagnosis of less than ten years. However, most studies failed to support any association between age and BRAFV600E [12,13,38]. It seems reasonable to hypothesize that, in studies including more adolescents than children, the prevalence increases, to “catch up” with that series of adult patients only. It is questionable whether this significantly different frequency of BRAF V600E between pediatric and adult populations may be implicated in their distinct clinicopathological characteristics and prognosis.

The gender distribution among PTC patients presents a well-known dimorphism, both in adults and in children or adolescents. Girls are more frequently diagnosed with PTC than boys by a ratio of 4:1. On the other hand, the BRAF V600E mutation is found more prevalent in male PTC patients. However, the relationship between BRAF V600E mutation and gender did not reach the level of statistical significance in this pooled data (*p* = 0.06). This finding is in accordance with the results of several other protocols [17,18,20,37,38,42,45,52]. Only two studies describe an association of the BRAF V600E mutation with the male gender [7,12]. It seems clear that the great female predominance among PTC patients in all age groups, opposed to the association of the BRAF V600E mutation with male gender in some studies, cannot be attributed to this genetic alteration according to the currently available data. 

Regarding the intrinsic morphological features of a tumor, the diametrical tumor size, expressed in metric data, was not found to be significantly associated with a mutated BRAF variant. Our finding is in accordance with the vast majority of studies investigating the relationship between the presence of BRAFV600E mutation presence and tumor size [12,13,17,18,20,37,42,45]. Sisdelli et al. [46] supported an association of BRAF V600E mutation with larger tumor diameter, while Li et al. found the BRAFV600E mutation more frequent in patients with smaller tumor size [7]. Tumor size is the first element that guides the management algorithm of a thyroid nodule management and, thus, appears as an important variable in the risk stratification of a PTC by TNM grading. Diameters larger than 1 cm are defined as the precursor for a more aggressive oncogenic behaviour [1,21], independently of the BRAF V600E mutation. It is obvious that differences in the reporting of tumor size over time are significantly correlated with the improvement of ultrasound diagnostic ability, combined with higher qualifications and awareness among health professionals.

Multifocality, as another aggressive locoregional prognostic factor, was not found to be associated with BRAF gene mutation. Eleven studies were included in the meta-analysis and the data are not only scarce but also divergent. Our results are in agreement with those of three studies [17,45], while two other studies found a negative correlation with BRAFV600E mutations identified more frequently in patients lacking multifocal tumor [7,18]. Only in the study by Onder et al. was BRAFV600E mutation more frequently present in cases with multifocal tumors [38], while Şenyürek et al. [45] reached a borderline positive association (*p* = 0.052). 

Turning to parameters indicative of extra-thyroid disease, we did not demonstrate any association of BRAF gene mutation with vascular invasion, as did other researchers [12,13,17,42]. High quality data on the effect of BRAF gene mutation on vascular invasion is lacking in the literature, perhaps due to the limited recording of vascular invasion as an independent prognostic parameter by investigators. ETE did not exhibit any difference in rate based on the presence or absence of the BRAF mutation. Similarly, previous studies reported no effect of BRAF V600E alteration on ETE [13,17,37,38,42]. Paradoxically, Geng et al. reported a negative correlation of BRAF V600E variant with ETE [18]. Furthermore, BRAF V600E mutation status was not associated with LNM. According to our results, most studies failed to support any association [13,17,18,20,38,42]. BRAF V600E was significantly more frequent in the BRAF V600E positive PCT patients, studied only by Li et al. and Oishi et al. [7,37].

Finally, in addition to the extent of the disease at diagnosis, another important parameter that determines long-term outcome is tumor recurrence. However, only one study described a positive association between BRAF mutation status and risk for recurrence [38]. The absence of an association between tumor recurrence and mutated BRAF is also only supported by scarce data [17,18]. Pooling of the available evidence in this study did not confirm any relevance between recurrence and BRAF mutations, due to very few and heterogenous data. 

In the present meta-analysis, mutated BRAF was significantly associated with the presence of distant metastasis at diagnosis. Distant metastasis at diagnosis is usually considered an indicator of rapid growth of the primary tumor and a consistent reflection of poor disease prognosis. Data regarding the relation between BRAF mutation and the presence of distant metastasis are reported to be largely contradictory in the literature for both children and adults. In the majority of the cohorts investigated in the present meta-analysis, the prevalence of distant metastasis at diagnosis was low, as the total metastatic PCT events at diagnosis were pooled at the raw number of only 120 individuals. 

According to our finding, the probability of detecting a mutated BRAF allele at diagnosis was significantly lower (OR:0.316) among patients with distant metastasis compared with those without metastatic disease. In adult PCT, it has been demonstrated by meta-analyses that BRAF mutation is also emerging as negatively associated with the presence of metastasis, but without reaching significance [10]. The validity of our finding is further firmly supported by the fact that heterogeneity was not evident in the pool of the available pediatric data (0%). It is highly significant that an emerging value for the molecular status BRAF is apparent, in terms of predicting the clinical course of PCT in children and adolescents. 

An end point in the management of patients with PCT is the assessment of long-term survival, even in the pediatric population. The positive or negative effect of a prognostic factor ideally reflects the survival rate. The analysis of survival rates as a mathematic outcome is characterized by a specific burden, in order to obtain a reliable to estimate when based on retrospective data. The population subject to “loss of follow-up” during transition to adult health care professionals also complicates the derived survival outcomes, as systematic, long-term follow-up of pediatric patients with PTC is prone to missing data, especially when research in conducted out of a registry. In the available literature, the overall survival rate is estimated ≥98.5% and reached 100% in the four studies that conducted follow-up for 5.8–19.5 (4.5–52.8), 5.5–38.8, 10–42 and 6 years, respectively [12,20,34,36]. Interestingly, Hanke et al. did not find any difference in progression-free survival (PFS) at 10 years based on BRAF V600E mutational status [12].

Furthermore, Nies et al. observed that extrapulmonary metastatic disease was recorded in all cases that died, hypothesizing that BRAF V600E positive patients had smaller tumor sizes and a delayed diagnosis of metastasis due to the poor sensitivity of radioactive iodine (RAI) scans [36]. However, it is noteworthy that, even in the presence of metastatic disease, in a large series of 1433 pediatric patients with PTC, long-term follow-up has shown 5-, 15-, and 30-yr survival rates of 98%, 97%, and 91%, respectively [53]. It is obvious that future high quality cohort data, through registry implementation, could longitudinally address the question of the association between BRAF status and distinct survival rates among pediatric PTC survivors.

The meta-analytic approach to adult PTC data [6,7,8,9,10] has reached conclusions which are not parallel to the findings of the present study and, thus, may seem unexpected. It is noteworthy that, in the present analysis, BRAF mutation was not associated with potential aggressive prognostic factors or the overall survival rate of pediatric PTC patients, except for the presence of distant metastasis. In contrast to our findings, adult data suggest that the presence of BRAF V600E is significantly associated with a cluster of tumor prognostic factors (tumor diameter, lymph node metastasis, multifocality, vascular invasion and extra-thyroid extension) [10]. Applying the rationale that children “are not just small adults”, this study provides evidence that BRAF gene analysis could also be applied during childhood PCT, as a marker for the prognosis of distant metastatic disease.

Thus, this meta-analysis could support the hypothesis that BRAF mutation status may provide part of the explanation for the different biomolecular behaviour of PTC in adult and pediatric populations. Moreover, it also justifies the increased interest in the study of fusions that are found to be more prevalent and associated with aggressive potential and unfavorable events. Franco et al. found that patients with RET/NTRK fusions had exhibited worse outcomes than those with BRAF-mutant disease [27]. Even BRAF fusions, reported in 2.7% of PTC pediatric cases [4], were associated with younger age [46] and aggressive disease, as implied by more frequent ETE, LNM and DM, as well as with requirement for higher RAI treatment doses [40]. 

Although this meta-analysis included 37 studies, and a total of 1799 PTC pediatric patients with PTC, to investigate all PTC prognostic factors in relation to BRAF mutational status on risk stratification of pediatric patients, there were some limitations that should be acknowledged. The studies included populations of different demographic and racial characteristics, affected by a wide spectrum of environmental factors, and who received a variety of methods of diagnosis and molecular analysis. All these parameters increased the heterogeneity of the sample and reflected the burden of drawing firm conclusions in mathematical random effects models. In addition, several studies were performed in a small number of patients, analysing only some of the outcomes reviewed here. Furthermore, the present study did not analyze different therapeutic strategies in PTC and the evaluation of different treatment approaches in pediatric and adolescent PTC was out of the scope of the present protocol. Finally, most of the data meta-analyzed in the present study originated from cross-sectional and retrospective previous studies, thus complicating the ability to demonstrate causality.

## 5. Conclusions

In conclusion, BRAF V600E mutation is less common in children and adolescents than in adults. Its prognostic potential lies in its significant negative relationship with the presence of distant metastasis. No significant correlation between BRAF mutational status and gender, tumor size, multifocality, lymph node metastasis, extrathyroidal extension, vascular invasion, tumor recurrence or survival rate is evident among children and adolescents with PTC. Further research is needed in order to describe in more detail its role in the risk stratification and management of pediatric and adolescent patients with PTC, and to establish guidelines. However, it remains a target for molecular therapy and immunomodulation with BRAF inhibitors. 

## Figures and Tables

**Table 1:** Included studies’ characteristics.

No	Authors, Year	Study Type	Recruitment (Country, Time)	Sample Size (*n*)	Sample Origin	Age (Years)	Gender (Boys/Girls)	BRAF Mutations Prevalence (%)	
								BRAF V600E	Other
1	Alzahrani [17], 2017	RC	Middle East, 1998–2015	79	registry	8–18	11/68	24	
2	Ballester [24], 2016	RCrS	USA, 2009–2014	25	clinical	10–19	6/19	40	
3	Buryk [25], 2013	RCaS	USA, 2009–2012	5	clinical	12–15	1/4	40	
4	Cordioli [16], 2017	RC	Brazil, NR	35	clinical	4–18	9/26	8.6	
5	Espadinha [26], 2009	C	Portugal, 2000–2007	15	clinical	5–21	4/11	7	
6	Franko [27], 2022	RC	USA, 1989–2019	122	clinical	<18	NR	21.3	0.75 (T599del)
7	Geng [18], 2017	RC	China, 1994–2014	48	clinical	3–14	19/29	35.4	
8	Gertz [15], 2016	RCrS	USA, 2008–2012	14	registry	8–18	5/9	31	7 (c.1799_1801delTGA)
9	Givens [13], 2014	RCrS	USA, 1999–2012	19	registry	3–18	NR	36.8	
10	Hardee [20], 2017	RCrS	USA, 2003–2015	50	registry	<21	15/35	48	
11	Henke [12], 2014	RCrs	USA, 1973–2005	27	registry	6–21	6/21	63	
12	Hess [28], 2022	RCrS	USA, 2010–2019	27	clinical	9.1–18.7	4/23	33.3	
13	Kumagai [11], 2004	C	Japan/Ukraine, 1962–1995	44	registry	<17	NR		6.81 (T1796A)
14	Kure [29], 2019	RC	Japan, 2009–2017	14	registry	13–21	0/14	14.3	
15	Kurt [30], 2012	C	Turkey, 1995–2010	2	registry	14–20	1/1	50	
16	Lee [31], 2021	RCrS	Korea, 1983–2020	106	clinical/registry	4.3–19.8	22/ 84	38.7	
17	Li [7], 2022	RC	China, 2018–2021	169	clinical	6–18	40/129	57.4	
18	Macerola [32], 2021	RC	Italy, 2014–2020	163	registry	8–18	47/116	36.2	0.6 (K599I)
19	Mitsutake [33], 2015	RC	Japan, 2013–2014	67	clinical	9–22	NR	64.2	
20	Mollen [34], 2022	RCrS	USA, 2001–2017	62	clinical	4.2–18.9	47/15	30.6	
21	Mostufi-Moab [19], 2018	RCrS	USA, 1989–2012	62	registry	2–18	NR	19.4	
22	Newfield [35], 2022	RC	USA, 2001–2015	39	registry	<18	NR	28.2	2.6 (K601E)
23	Nies [36], 2021	RC	USA, 1946–2019	94	registry	10–16	NR	8.5	
24	Oishi [37], 2017	CC	Japan, 1991–2013	81	registry	6–20	7/74	54	
25	Onder [38], 2016	RC	Turkey, 1995–2015	50	registry	6–18	9/41	30	
26	Passon [39], 2015	RC	Italy, NR	2	clinical	17–19	0/2	0	
27	Pekova [40], 2019	RC	Czech Rep, 2003–2017	83	clinical	14.2 ± 3.4	24/59	18.1	
28	Pessôa-Pereira [41], 2019	RC	Brazil, 2006–2012	5	registry	12–20	0/5	20	
29	Poyrazoglu [42], 2017	RC	Turkey, 1983–2015	75	clinical	1.3–17.8	24/51	25	
30	Prasad ML [14], 2016	RCrS	USA, 2009–2015	28	clinical	6–18	8/20	48	
31	Rogounovitch [43], 2021	RC	Belarus, 2001–2007	34	registry	4–14	12/22	14.7	0 (K601E)
32	Romittii [44], 2012	RCrS	Brazil, NR	3	registry	10–18	0/3	0	
33	Şenyürek [45], 2022	RC	Turkey, 1995–2020	55	registry	5–18	15/55	33	
34	Sisdeli L [46], 2019	RC	Brazil, 1993–2017	80	registry	<18	NR	15	
35	Stenman [47], 2021	RC	Sweden, 1992–2021	5	registry	9–15	2/3	20	
36	Vasko V [48], 2005	RCrS	Ukraine, 1999–2004	4	clinical	14–20	2/2	25	
37	Zou M [49], 2014	RC	Saudi Arabia, 1987–2006	6	clinical	12–21	1/5	16.7	

NR: not reported, RC: retrospective cohort, C: cohort, RCaS: retrospective case series, RCrS: retrospective cross sectional, CC: case control. Age in presented as min-max or mean ± standard deviation.

**Table 2:** Tumor Characteristics among PTC patients according to BRAF analysis.

No	Author, Year	Total Study Sample (*n*)	BRAF Mutation Status (+/−)	Sample per BRAF Group (*n*)	Tumor Size (cm) or (f*)	Multifocality (%)	Vascular Invasion (%)	LNM (%)	ETE (%)	DM (%)	Tumor Recurrence (%)
1	Alzahrani [17], 2017	79	+	19	2.8 ± 1.4	50	40	86.7	35.7	0	52.6
			−	60	3.3 ± 1.6	53.8	51.4	82.8	46.2	15	33.9
2	Ballester [24], 2016	25	+	10	NR	NR	NR	50	NR	NR	NR
			−	15	NR	NR	NR	46.7	NR	NR	NR
3	Buryk [25], 2013	5	+	2	2.7 ± 0.56	NR	NR	100	NR	NR	NR
			−	3	1.7 ± 0.17	NR	NR	33.3	NR	NR	NR
4	Cordioli [16], 2017	35	+	3	4.6 ± 1.25	NR	NR	100	0	0	NR
			−	16	2.9 ± 1.48	NR	NR	88.2	64.7	35.2	NR
5	Espadinha [26], 2009	15	+	1	NR	NR	NR	0	NR	NR	NR
			−	14	NR	NR	NR	NR	NR	NR	NR
6	Franko [27], 2022	122	+	26	* <2 cm = 11, 2–4 cm = 5, >4 cm = 10	NR	30.7	72	46.1	0	NR
			−	96	NR	NR	NR	NR	NR	NR	NR
7	Geng [18], 2017	48	+	17	* <2 cm = 2, 2–4 cm = 11, >4 cm = 4	20	NR	64.7	16.0	36.6	20
			−	31	* 2–4 cm = 17, >4 cm = 14	80	NR	80.6	84.0	63.4	80
8	Gertz [15], 2016	14	+	4	1.7 ± 1.2	NR	33.3	NR	25	0	NR
			−	9	2.7 ± 2.4	NR	33.3	NR	22.2	0	NR
9	Givens [13], 2014	19	+	7	2.08 ± 1.21	NR	NR	NR	60	0	16.7
			−	12	2.22 ± 1.78	NR	NR	NR	62.5	41.7	12.5
10	Hardee [20], 2017	50	+	24	* <2cm = 18, 2–4 cm = 2, >4 cm = 4	NR	NR	58	0	NR	21
			−	26	* <2cm = 13, 2–4 cm = 5, >4 cm = 7	NR	NR	69%	4	NR	8
11	Henke [12], 2014	27	+	17	NR	NR	NR	64.7	70.6	5.9	NR
			−	10	NR	NR	NR	60	50	0	NR
12	Hess [28], 2022	27	+	9	1.37 ± 1.09	NR	NR	42.8	NR	NR	NR
			−	18	3.22 ± 2.04	NR	NR	68.75	NR	NR	NR
13	Kumagai [11], 2004	44	+	3	1.56 ± 0.87	NR	NR	33.3	NR	0	NR
			−	NR	NR	NR	NR	NR	NR	NR	NR
14	Kure [29], 2019	14	+	2	1.25 ± 0.77	NR	50	33.3	0	0	NR
			−	12	2.34 ± 1.79	NR	50	66.6	8.3	8.33	NR
15	Kurt [30], 2012	2	+	1	NR	NR	NR	100	100	0	NR
			−	1	NR	32.5	NR	0	0	0	NR
16	Lee [31], 2021	106	+	41	1.40 ± 1.00	41.5	NR	68.4	60.5	2.5	16.2
			−	65	2.10 ± 1.30	23.7	NR	76.2	75.8	43.18	46.6
17	Li [7], 2022	169	+	97	1.55 ± 1.03	50	NR	14.4	24.7	2.1	2
			−	72	2.49 ± 1.18	NR	NR	8.3	36.1	4.1	8.3
18	Macerola [32], 2021	163	+	59	NR	NR	NR	NR	NR	NR	NR
			−	104	NR	NR	NR	NR	NR	NR	NR
19	Mitsutake [33], 2015	67	+	43	1.22 ± 0.68	NR	NR	14.2	58.1	0	NR
			−	20	1.83 ± 0.95	NR	NR	20	35	10.5	NR
20	Mollen [34], 2022	62	+	19	NR	NR	NR	NR	NR	NR	NR
			−	43	NR	NR	NR	NR	NR	NR	NR
21	Mostufi-Moab [19], 2018	62	+	12	1.10–4.00	NR	NR	63.6	NR	0	NR
			−	50	NR	NR	NR	NR	NR	NR	NR
22	Newfield [35], 2022	39	+	11	2.67 ± 1.98	NR	54.5	81.8	NR	0	NR
			−	18	2.70 ± 1.44	NR	50	50	NR	7.14	NR
23	Nies [36], 2021	94	+	8	2.90 (2.3–3.2)	NR	NR	100	NR	100	NR
			−	86	3.50 (2.3–5.5)	NR	NR	NR	NR	100	NR
24	Oishi [37], 2017	81	+	44	3.20 ± 1.8	NR	NR	98	36	0	NR
			−	37	2.80 ± 1.3	NR	NR	81	44	8	NR
25	Onder [38], 2016	50	+	15	2.12 ± NR	93.3	NR	60	13.3	0	33.3
			−	35	2.26 ± NR	57.14	NR	61.5	8.57	14.2	5.7
26	Passon [39], 2015	2	+	0	NR	NR	NR	0	NR	0	NR
			−	2	NR	NR	NR	0	NR	0	NR
27	Pekova [40], 2019	83	+	15	2.00 ± 1.06	53.3	20	46.6	40	0	20
			−	68	2.22 ± 1.36	55.8	24.3	76.47	54.4	14.7	8.8
28	Pessôa-Pereira [41], 2019	5	+	1	1 ± 0	0	0	0	0	0	NR
			−	4	2.32 ± 1.39	75	25	50	0	0	NR
29	Poyrazoglu [42], 2017	75	+	14	* ≤1 cm = 3 >1 cm = 11	85.7	50	57.1	42.8	7.1	NR
			−	42	* ≤1 cm = 16 >1 cm = 26	42.8	40.5	38	28.6	9.5	NR
30	Prasad ML [14], 2016	28	+	13	1.44 ± 1.04	23.1	23.1	38.4	7.7	0	NR
			−	14	2.21 ± 1.13	50	NR	71.4	NR	14.3%	NR
31	Rogounovitch [43], 2021	34	+	5	1.44 ± 0.34	0	100	100	0	0	NR
			−	29	1.6 ± 0.9	NR	NR	NR	NR	NR	NR
32	Romittii [44], 2012	3	+	-	-	-	-	-	-	-	-
			−	1	10.5 ± 0	NR	NR		NR	0	NR
33	Şenyürek [45], 2022	55	+	18	1.50 (0.6–5)	83.3	55.5	33.3	25	0	33.3
			−	37	1.40 (0.4–5)	56.7	32.4	35.1	21.6	8.1	2.7
34	Sisdeli L [46], 2019	80	+	12	3.35 ± 1.38	NR	NR	75	NR	25	NR
			−	68	2.64 ± 1.58	NR	NR	NR	NR	NR	NR
35	Stenman [47], 2021	5	+	1	4.20 ± 0	0	NR	100	100	NR	100
			−	4	4.57 ± 2.12	50	NR	100	75	25	50
36	Vasko V [48], 2005	4	+	3	2.36 ± 0.55	0	NR	NR	NR	NR	NR
			−	1	1.50 ± 0	0	NR	NR	NR	NR	NR
37	Zou M [49], 2014	6	+	1	NR	NR	NR	0	NR	0	NR
			−	3	NR	NR	NR	44.4	NR	0	NR

NR: not reported, LNM lymph node metastasis, ETE extra-thyroid extension, DM distant metastasis. Tumor size data are presented as mean ± standard deviation or median (min–max) in most included studies. * Tumor size data presented as categorical variable (f = number of participants per tumor size group).

## Data Availability

The data used to support the findings of this study are publicly available and listed in the supplementary material of this article.

## Supplementary Materials

The following supporting information can be downloaded at: https://www.mdpi.com/article/10.3390/diagnostics13061187/s1, Table S1: Included studies characteristics regarding methods of molecular analysis and funding sources/competing interest.
