# Supplementary material for: The Prognostic Significance of BRAF Gene Analysis in Children and Adolescents with Papillary Thyroid Carcinoma: A Systematic Review and Meta-Analysis
Source: Diagnostics (Basel). 2023 Mar 21;13(6):1187. doi: 10.3390/diagnostics13061187 (PMC10047331; doi:10.3390/diagnostics13061187)
Supplement: Supplementary file 1 [file diagnostics-13-01187-s001.zip › diagnostics-2276014-supplementary.pdf]

Supplement Table S1. Included studies characteristics regarding methods of molecular analysis and funding sources/ competing interest.

No	Authors, Year	Follow-Up Duration (years)	Method of BRAF analysis	Funding Sources & Authors conflict of Interest
1	Alzahrani[17], 2017	1.2-16	PCR/ Direct sequencing	This research did not receive any specific grant from any funding agency in the public, commercial or not-for-profit sector.
2	Ballester[24], 2016	NR	RT PCR assay	NR
3	Buryk[25], 2013	NR	PCR and post-PCR fluorescence melting curve analysis	No conflict of interest to disclose.
4	Cordioli[16], 2017	NR	PCR sequencing	Research grants 2013/03867-5 and 2015/60330-8 from The São Paulo State Research Foundation (FAPESP) and grant 470441/2013-5 The São Paulo State Research Foundation (FAPESP) and grant 470441/2013-5 The São Paulo State Research Foundation (FAPESP) and grant 470441/2013-5 from The Brazilian Research Council (CNPq). LM and AUB are recipients of fellowships from FAPESP. JMC is a recipient of a scholarship of Research Productivity from CNPq
5	Espadinha[26], 2009	NR	automatic sequencing of PCR products (QRT-PCR/ direct sequencing)	NR
6	Franco[27], 2022	NR	sequencing	Supported in part by a grant from NIH R01CA214511 (A.T.F.), The Children's Hospital of Philadelphia Frontier Programs (A.T.F., J.C.R.-F., A.I., S.M.-M., L.S., E.R., and A.J.B.), and the Children's Hospital of Philadelphia Foerderer Grant (J.C.R.-F.).
7	Geng[18], 2017	NR	qRT-PCR	The author(s) declare that they have no conflict of interest. This work was supported in part by Beijing Munic- ipal Administration of Hospitals Clinical Medicine Development of Special Funding Support (ZYLX201508), Beijing Municipal Science and Technology Project (D131100005313014), and Beijing Health System Top Level Health Technical Personnel Training Plan (20153079).
8	Gertz[15], 2016	2.6 ±1.58	Sanger sequencing	The authors wish to thank the University of Wisconsin. Translation Research Initiatives in Pathology Laboratory, in part supported by the University of Wisconsin Department of Pathology and Laboratory Medicine, in Madison, and University of Wisconsin Carbone Cancer Center grant P30 CA014520, for use of its facilities and services. Funding was provided by the Department of Pathology and Laboratory Medicine at the University of Wisconsin, Madison.
9	Givens[13], 2014	NR	pyrosequencing PCR	The authors have no funding, financial relationships, or conflicts of interest to disclose

10	Hardee[20], 2017	1-12	PCR primers, single strand conformational polymorphism (SSCP)	The author(s) received no financial support for the research, author- ship, and/or publication of this article. The author(s) declared no potential conflicts of interest with respect to the research, authorship, and/or publication of this article.
11	Henke[12], 2014	5.5–38.8	restriction fragment length polymorphism (RFLP) analysis ,PCR	NR
12	Hess[28], 2022	10	next generation sequencing analysis	No competing financial interests exist. This study was supported by a Learner's Research Fund grant at Phoenix Children's Hospital awarded to Jennifer Hess.
13	Kumagai[11], 2004	NR	PCR/ direct sequencing	This work was supported by Grants-in-Aid for Scientific Research 13671158, 14380256, 14406017, and 15590981 and the 21st Century COE Program "International Consortium for Medical Care of Hibakusha and Radiation Life Science" from the Ministry of Education, Culture, Sports, Science, and Technology
14	Kure[29], 2019	1-8	real-time PCR using allele specific primers	This study was funded by the Japanese Association of University Women [the 66th Homes Scholarship, 2014]; and the Children's Cancer Association of Japan [2017–2019]. The authors declare that there is no conflict of interest.
15	Kurt[30], 2012	NR	restriction fragment length polymorphism (RFLP)	The authors would like to thank to Scientific and Technological Research Council of Turkey (TUBITAK) for their support (Grant No: 19816)
16	Lee[31], 2021	0.3-37.3	BRAF IHC or sequencing	The Ministry of Science, ICT and Future Planning (NRF-2016R1A2B4012417 and 2019R1A2C2084332); the Korean Ministry of Health and Welfare (H14C1277); the Ministry of Education (2020R1A6A1A03047972); and the SNUH Research Fund (04-2015-0830).
17	Li[7], 2022	0.08-4	real-time PCR using allele specific primers	NR
18	Macerola[32], 2021	NR	real time PCR and high-resolution melt analysis	Reagents and consumables were obtained with funds from the University of Pisa
19	Mitsutake[33], 2015	NR	direct DNA sequencing	"This work was supported in part by JSPS KAKENHI, grant number: 25461989 (TF), 25257508 (SY), 26293222 (SY), 26293142 (NM)."
20	Mollen[34], 2022	0.04-18	targeted amplification based next generation sequencing	No competing financial interests exist. Funding was provided from the UPMC Children's Hospital of Pittsburgh Scientific Program Fund.
21	Mostufi-Moab[19], 2018	NR	multiplex PCR for gene mutations	National Cancer Institute (K07 CA166177; S.M.M.)
22	Newfield[35], 2022	0.5	real-time PCR using allele specific primers and probes	This research did not receive any specific grant from funding agencies in the public, commercial, or not-for-profit sectors. WJ, EL, RON, and RSN have nothing to disclose. DXS is an employee of Quest Diagnostics, and FMH is a former employee of Quest Diagnostics, and other than some equity interest in the company, they do not have other conflicts of interest.
23	Nies[36], 2021	0.8-66.1	DNA sequencing	Marloes Nies received funding from the Junior Scientific Master Class Groningen, the Prince Bernhard

				Culture Fund, and the Academy Ter Meulen Grant of the Royal Netherlands Academy of Arts and Sciences for her research at The University of Texas MD Anderson Cancer Center. This work was supported in part by the National Institutes of Health/National Cancer Institute Cancer Center Support Grant (award No. P30CA016672) and used the Biostatistics Resource Group.
24	Oishi[37], 2017	NR	allele-specific- PCR and/or Sanger sequencing	This study was funded by Japan Society for the Promotion of Science (JSPS) KAKENHI (Grant Number 25293087 and 90,623,661). The authors declare that they have no conflict of interest.
25	Onder[38], 2016	1.08-20.8	allele specific real-time PCR- based direct sequencing	All authors declare that they have no conflict of interest. Funding: NR
26	Passon[39], 2015	NR	PCR- based direct sequencing	Associazione Italiana per la Ricerca sul Cancro (AIRC) (Project No IG 10296), to DR (MIUR: Grant 2010NFEB9L_003), and to MC (MIUR: Grant RBFR12FI27_003). The authors declare that they have no conflict of interest.
27	Pekova[40], 2019	0.08-14	next generation sequencing	This work was supported by the Ministry of Health of the Czech Republic. AZV (16-32665A) grants. The authors declare that there is no conflict of interest that could be perceived as prejudicing the impartiality of the research reported.
28	Pessôa-Pereira[41], 2019	NR	direct DNA sequencing	Grants of FAPESB (Fundação de Amparo à Pesquisa no Estado da Bahia, APP0040/2011). No potential conflict of interest relevant to this article was reported.
29	Poyrazoglu[42], 2017	1-14.1	pyrosequencing	Conflict of interest: None declared.The authors declared that this study received no financial support.
30	Prasad ML[14], 2016	0.25-6.9	single-strand conformational polymorphism, next-generation sequencing	This study was supported by the authors' intradepartmental funding. Manju L. Prasad and Yuri E. Nikiforov received an honorarium from Loxo Oncology, the maker of tyrosine receptor kinase inhibitor, for their participation in an advisory meeting. Yuri E. Nikiforov reports consulting fees from Quest Diagnostics outside the submitted work.
31	Rogounovitch[43], 2021	NR	direct DNA sequencing	Belarusian Republican Foundation for Fundamental Research (BRFFR), grant NoБ08MC-007, the Institute of Physiology, National Academy of Sciences of Belarus (Minsk, Belarus), the Japan Society for the Promotion of Science (JSPS), KAKENHI Grant Number 19KK02670001, and intramurally by the Atomic Bomb Disease Institute, Nagasaki University
32	Romittii[44], 2012	NR	RT-PCR, direct sequencing	This work was supported by Co-ordenac¸a˜o de Aperfei- c¸oamento de Pessoal de N´ıvel Superior (CAPES), Conselho Nacional de Desenvolvimento Cient´ıfico e Tecnolo´gico (CNPq), Fundac¸a˜o de Apoio a Pesquisa do Rio Grande do Sul (FAPERGS), and Fundo de Incentivo a Pesquisa do Hospital de Cl´ınicas de Porto Alegre (FIPE), Brazil. The authors declare that

				there is no conflict of interest that could be perceived as prejudicing the impartiality of the re- search reported.
33	Şenyürek[45], 2022	0.16-1.8	pyrosequencing	The authors declared that this study received no financial support
34	Sisdeli L[46], 2019	NR	allele specific real-time PCR- based direct sequencing	FAPESP, grant number 2014/06570-6 . J.M.C. is a recipient of a scholarship of Research Productivity from CNPq - G.A.C.-G. is beneficiary of a scholarship from Coordenação de Aperfeiçoamento de Pessoal de Nível Superior (CAPES). L.S. and L.M. are beneficiaries of a scholarship from FAPESP. The authors declare that there is not coflict of interest.
35	Stenman[47], 2021	NR	whole genome sequencing	Grant support from the Swedish Cancer Society, the Swedish Society for Medical Research and Karolinska Institutet. The authors declare that there is no conflict of interest
36	Vasko V[48], 2005	NR	PCR direct sequencing	This study was supported by a research grant from the Flight At- tendant Medical Research Institute and a Johns Hopkins Clinician Sci- entist Award (to M.X.)
37	Zou M[49], 2014	12	Sanger sequencing	The study is supported by a Biotechnology grant #10-BIO957-20 from King Abdulaziz City for Science and Technology.

NR: not reported
